# Solvent Vapor Treatment Effects on Poly(3-hexylthiophene) Thin Films and its Application for Interpenetrating Heterojunction Organic Solar Cells

**DOI:** 10.3390/ma3114939

**Published:** 2010-11-15

**Authors:** Tetsuro Hori, Varutt Kittichungchit, Hiroki Moritou, Hitoshi Kubo, Akihiko Fujii, Masanori Ozaki

**Affiliations:** Division of Electrical, Electronic and Information Engineering, Graduate School of Engineering, Osaka University, 2-1 Yamada-oka, Suita, Osaka 565-0871, Japan

**Keywords:** solvent vapor treatment, interpenetrating heterojunction, organic solar cells, poly(3-hexylthiophene), fullerene

## Abstract

The solvent vapor treatment (SVT) for poly(3-hexylthiophene) (PAT6) films and its application to interpenetrating heterojunction organic solar cells have been studied. It was found that SVT could improve the crystallinity and electrical characteristics of the PAT6 films. We fabricated organic solar cells with an interpenetrating structure of PAT6 and fullerenes utilizing the SVT process, and discuss the improved performance of the solar cells by taking the film crystallinity, optical properties, and morphology into consideration.

## 1. Introduction

Since the discovery of the suppression of photoluminescence [1], photoinduced electron transfer [2], and enhancement of photoconductivity [3,4] between the π-conjugated main chain in conducting polymers and C_60_, conducting polymer-fullerene systems have been investigated as donor-acceptor type organic solar cells. Two different typical device structures—a conducting polymer-C_60_ composite structure and a layered structure—have mainly been studied in the field of organic thin-film solar cells. Semilayered structures with an interpenetrating interface have also been proposed as a candidate structure exhibiting high photovoltaic conversion efficiency [5,6]. In the device structure with the interpenetrating interface, the photoinduced electron transfer occurs efficiently because of the large donor-acceptor interface area, and the generated electrons and holes can be effectively transported in n-type and p-type materials towards the electrodes, respectively. Recently, we have developed a simple, yet effective method of fabricating such donor-acceptor interpenetrating interfaces, and we reported an improvement in the photovoltaic conversion efficiency [7,8,9,10,11,12,13].

Electrical properties such as the carrier mobility and conductivity of organic thin films are important factors determining the energy conversion efficiency of organic solar cells. We reported on the crystallinity improvement of the C_60_ layer by substrate temperature control during C_60_ evaporation, resulting in the enhanced photovoltaic properties of interpenetrating heterojunction organic thin-film solar cells [13]. The crystallinity improvement of the conducting polymer layer, therefore, must also enhance the electrical properties of the conducting polymer layer and photovoltaic properties of the organic solar cells with an interpenetrating heterojunction structure.

Thin films of conducting polymers such as poly(3-hexylthiophene) (PAT6) are usually fabricated by spin-coating onto an appropriate substrate. Such a conducting polymer thin film fabricated by spin-coating is in an amorphous phase or has low crystallinity. Therefore, thermal annealing is usually adopted for controlling the crystallinity of polymer films [14,15]. Previously, it was reported that the crystallinity and alignment of a PAT6 thin film and its carrier mobility depended on the solubility and boiling point of the solvent used during spin-coating [16]. In our recent study, we reported initial findings on the solvent vapor treatment (SVT) for a PAT6 film fabricated by conventional spin-coating, which was effective for the improvement of the photovoltaic properties in the organic solar cells with an interpenetrating heterojunction structure [17]. The SVT might be an indispensable process for polymer film fabrication; however, detailed studies about SVT effects and the mechanism still remain to be clarified.

In this paper, we report a study on the SVT for a PAT6 spin-coated film, and we discuss its crystallinity and electrical characteristics. We report the application of SVT to fabricating organic solar cells with an interpenetrating interface structure, and the effects of SVT on the photovoltaic properties are discussed by taking the film crystallinity, optical properties, and morphology into consideration.

## 2. Experimental

**Figure 1:**
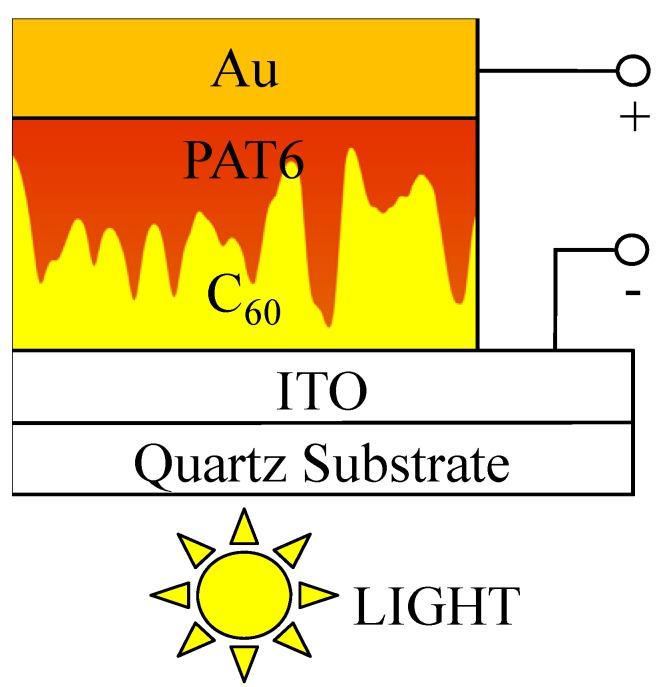
Typical structure of an interpenetrating heterojunction organic thin film solar cell.

PAT6 (Aldrich, Inc.) and C_60_ (Frontier Carbon, Ltd.) were used as purchased. Solar cells with the structure of ITO/C_60_/PAT6/Au were fabricated in the following way. C_60_ films with thicknesses of approximately 130 nm were deposited by thermal evaporation at an evaporation rate of 2.7–2.9 nm/min onto ITO-coated quartz substrates with a sheet resistance of 10 Ω/□ under a pressure of 10^−4^ Pa. The fabrication of the C_60_ films was carried out at a substrate temperature of 150 °C [13]. A chloroform solution of PAT6 of concentration 0.1 mol/L (16.6 mg/mL) was spin-coated onto the C_60_ layer to fabricate the C_60_/PAT6 interpenetrating layer, then the organic film was treated by SVT as described below. The total thickness of the active layers are approximately 300 nm [12]. An Au electrode was fabricated onto the PAT6 layer by thermal evaporation through a shadow mask under a pressure of 10^−4^ Pa. The evaporation rate of Au was approximately 1.0 Å/s. The thickness of the Au electrode was 70 nm. The active area of the solar cell was 1 × 2 mm^2^. In the cells, the ITO and the Au electrode collected electrons and holes, respectively. The typical structure of an interpenetrating heterojunction type organic thin-film solar cell is shown in Figure 1.

SVT was carried out in the following manner. Substrates with a PAT6 layer were set on a noncorrosive plate in a glass petri dish. Chloroform was injected into the petri dish, then the petri dish was sealed. The substrates were heated by a hot plate at a constant temperature for 20 min.

X-ray diffraction (XRD) patterns were measured using a Rigaku X-ray diffractometer (RINT 1100). The surface morphologies of the C_60_/PAT6 interpenetrating thin films were observed using a scanning electron microscope (SEM: Hitachi S-2100).

The current-voltage characteristics were measured with a high voltage source measurement unit (Keithley 237) under AM1.5 (100 mW/cm^2^) solar-illuminated conditions. From the current-voltage characteristics under AM1.5, the fill factor (FF) and energy conversion efficiency (η_e_) were estimated using the following definitions: FF = *I*_max_*V*_max_ / *I*_sc_*V*_oc_ and η_e_ = *I*_sc_*V*_oc_FF / P_in_, where *I*_max_ and *V*_max_ are the current and voltage at the maximum output power, *I*_sc_ is the short-circuit current density, *V*_oc_ is the open-circuit voltage, and P_in_ is the intensity of the incident light.

External quantum efficiency (EQE) spectra of solar cells were measured under the short-circuit condition using an electrometer (Keithley 617S) and light from a xenon lamp passing through a monochromator as a light source. The current-voltage characteristics and EQE spectra were then measured in vacuum at room temperature. The EQE was estimated using the following definition: EQE (%) = *I*_sc_ × 1,240/(λ (nm) × P_in_) ×1 00, where λ is the wavelength of incident light. The absorption spectra of the C_60_/PAT6 interpenetrating thin films on quartz substrates were measured using a spectrophotometer (Shimadzu UV-3150).

## 3. Results and Discussion

### 3.1. SVT Effects for spin-coated PAT6 thin films

**Figure 2:**
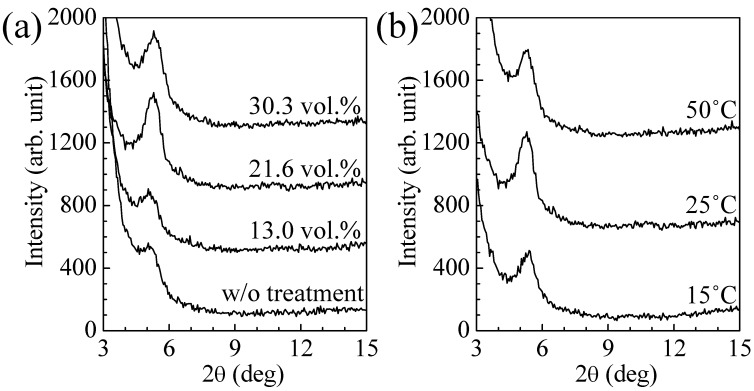
XRD patterns of the spin-coated PAT6 films depending on (**a**) vapor concentrations at constant temperature of 25 °C, and (**b**) temperature at at contant 21.6 vol % vapor concentration in SVT.

Figure 2 shows XRD patterns of the spin-coated PAT6 thin films after SVT with different vapor concentrations and temperatures. An increase in diffraction peak intensity at 2*θ* = 5.4° was observed at a vapor concentration of 21.6 vol % and vapor temperature of 25 °C. This diffraction peak is the first-order Bragg reflection, which corresponds to the interchain distance of the polymer main chain in the in-plane direction of the thiophene ring. The increase in the peak intensity indicates that the crystallinity of the spin-coated PAT6 thin film was improved for the in-plane stacking. The excess conditions of the SVT, such as higher vapor concentration or temperature, were not effective, which is due to a partial collapse of the main chain stacking structure.

**Figure 3:**
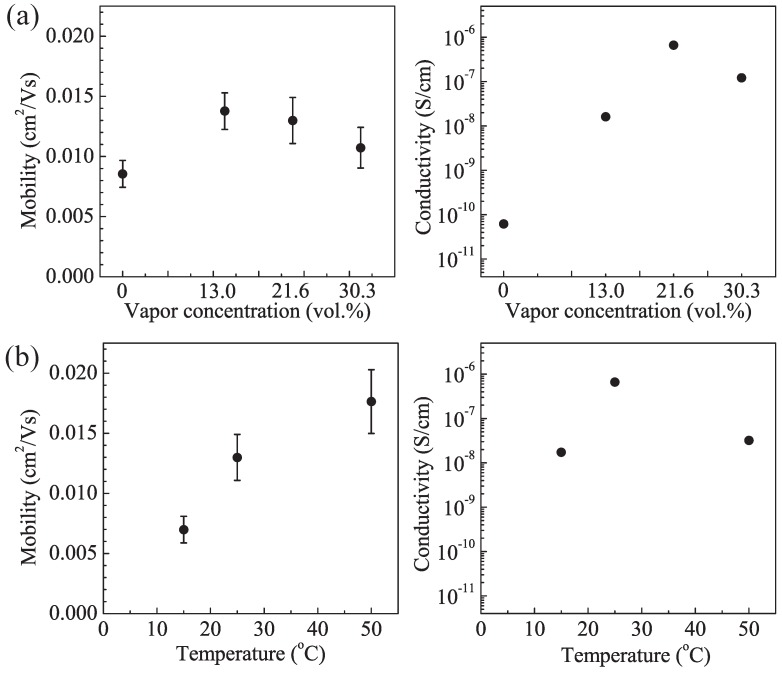
Hole mobility and conductivity of the PAT6 film depending on (**a**) concentrations at a temperature of 25 °C, and (**b**) temperature at 21.6 vol % vapor concentration in the SVT.

**Figure 4:**
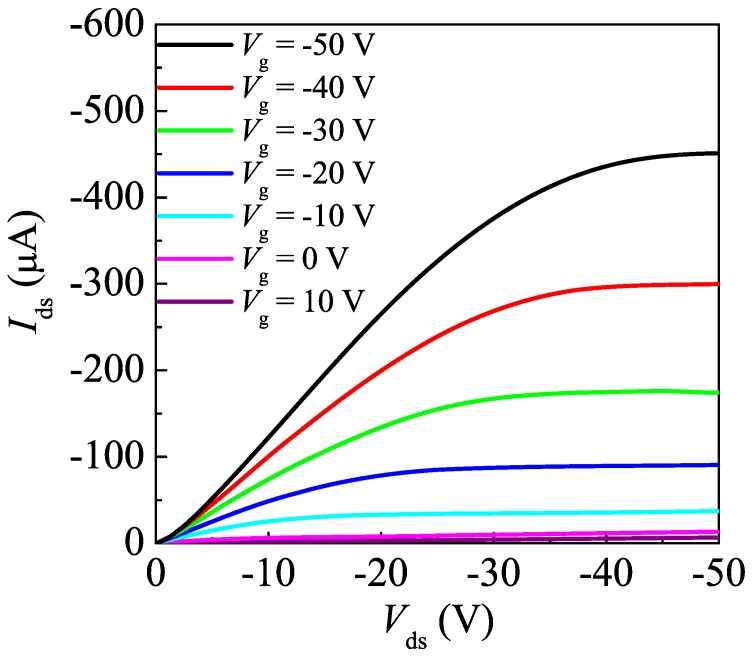
Typical result of field-effect transistor (FET) performance of the PAT6 layer after the SVT at 21.6 vol % concentration at 25 °C for 20 min.

Figure 3 shows the hole mobility and conductivity of the spin-coated PAT6 thin films after SVT with different vapor concentrations and temperatures. The hole mobility was estimated from the measured field-effect transistor (FET) characteristics of the spin-coated PAT6 thin film on an n-Si substrate with a bottom contact structure. For calculating the hole mobility μ, we used the following equation: *I*_ds_ = ½ × W/L × μC_ox_(*V*_G_-*V*_T_)^2^, where *I*_ds_, *V*_G_, and *V*_T_ are the source-drain current, gate voltage, and threshold voltage, respectively. W and L are the channel width and channel length of the FET, respectively. The typical result of FET performance of the PAT6 layer after the SVT at 21.6 vol % concentration at 25 °C for 20 min is shown in Figure 4. The conductivity was estimated from the measured current-voltage characteristics of ITO/PAT6/Au structured cells. For calculating the conductivity σ, we used the following equation: σ = 1/ρ = *I*/*V* × l, where ρ and l are the resistivity and thickness, respectively. The hole mobility and conductivity of the PAT6 thin film without SVT were 0.009 cm^2^/V·s and 6.2 × 10^−11^ S/cm, respectively. In the case of spin-coated PAT6 thin film with SVT at a vapor concentration of 21.6 vol % and vapor temperature of 25 °C, the hole mobility and conductivity were 0.013 cm^2^/V·s and 6.6 × 10^−7^ S/cm, respectively. Thus, the hole mobility and conductivity were enhanced using SVT by approximately a factor of 1.4 and by 4 orders of magnitude, respectively. The main chain and side chain of PAT6, which have high regioregularity, tend to be aligned parallel and perpendicular to the substrate under the film, respectively [18]. Therefore, the charge carriers in the PAT6 film efficiently move in the main chain direction and π-stacking direction, that is, intramolecular and intermolecular transport occur efficiently, respectively. It is considered that SVT is effective for improving the alignment of the main chain in the in-plane direction of the thiophene ring, whereas it is less effective for alignment in the π-stacking direction, the reason why the conductivity was measured in the direction perpendicular to the substrates whereas the hole mobility measured in the direction parallel to the substrates. In sandwich-type organic solar cell structures, charge carriers are transported in the direction perpendicular to the substrates, therefore, it is considered that SVT is an effective method for fabricating electronic devices containing a conducting polymer such as PAT6.

### 3.2. SVT Effect for the C_60_/PAT6 interpenetrating layer

**Figure 5:**
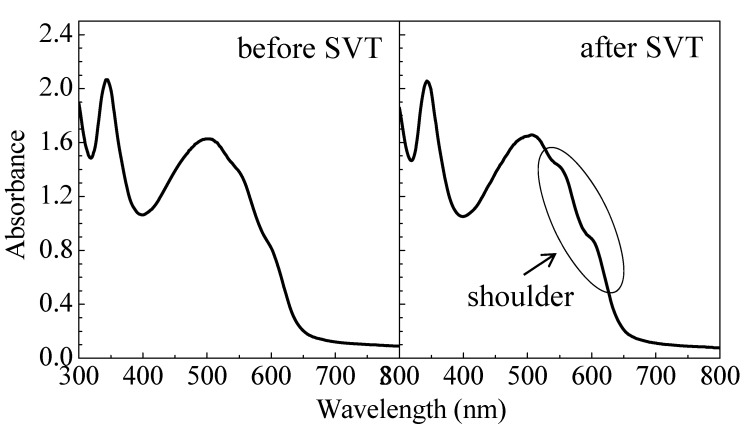
Absorption spectra of the C_60_/PAT6 interpenetrating layer before and after the SVT at 21.6 vol % concentration at 25 °C for 20 min.

Figure 5 shows absorption spectra of the C_60_/PAT6 interpenetrating layer before and after SVT with a vapor concentration of 21.6 vol % at 25 °C for 20 min. Although the spectral shape in the range of 300–500 nm, corresponding to C_60_ absorption, was unaffected by SVT, an absorption shoulder became remarkable at approximately 500–600 nm after SVT compared with before SVT. It is known that regioregular PAT6 has relatively high crystallinity and a long conjugation length in comparison with regiorandom PAT6. The peak at 510 nm corresponds to the π-π* energy transition of PAT6, and the shoulders at 560 and 610 nm correspond to the vibronic absorption from the regioregularity and the interplanar interaction of PAT6, respectively [19]. The appearance of these shoulders in the absorption spectrum indicates an improvement of the crystallinity and alignment of the PAT6 thin film [20]. Therefore, the crystallinity of PAT6 is improved by SVT in the C_60_/PAT6 interpenetrating layer as well as the spin-coated PAT6 thin film.

**Figure 6:**
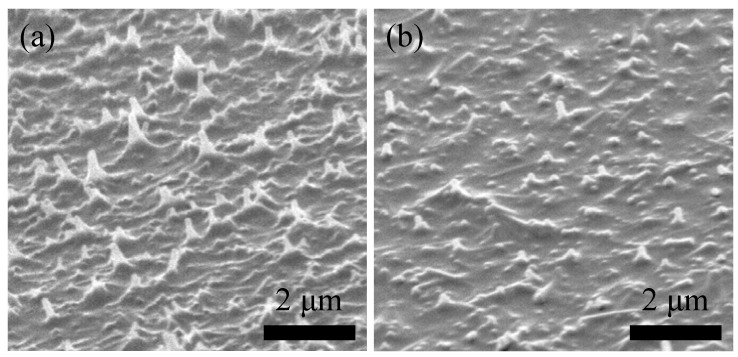
Scanning electron microscope (SEM) images of the C_60_/PAT6 interpenetrating layer (**a**) before and (**b**) after the SVT at 21.6 vol % concentration at 25 °C for 20 min.

The SEM image of the surface morphology of the C_60_/PAT6 interpenetrating layer without SVT is shown in Figure 6(a). The uneven morphology of the PAT6 film surface was confirmed, which was one of the reasons for the low *V*_oc_ in the photovoltaic characteristics because of the leak current between PAT6 and Au electrode [7,12,13]. As shown in Figure 6(b), the surface morphology of the C_60_/PAT6 interpenetrating layer after SVT was changed, resulting in lower roughness. Although the surface morphology of PAT6 thin film is not affected by SVT because the roughness of the PAT6 surface is flat, it is considered that the C_60_/PAT6 interpenetrating layer is planarized by SVT. Therefore, it is considered that higher *V*_oc_ and FF in the characteristics of solar cells could be expected upon SVT because of the improvement in the contact between PAT6 and the Au electrode.

### 3.3. Photovoltaic properties of the solar cells with SVT

**Figure 7:**
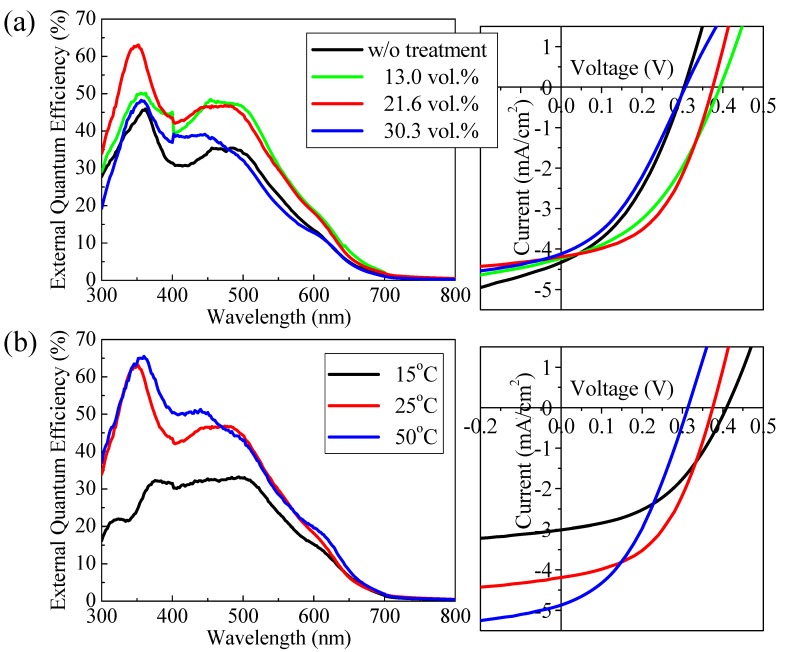
External quantum efficiency (EQE) spectra and current-voltage characteristics under AM1.5 (100 mW/cm^2^) solar-illuminated conditions in ITO/C_60_/PAT6/Au interpenetrating heterojunction solar cells depending on (**a**) vapor concentration at a temperature of 25 °C, and (**b**) temperature at 21.6 vol % vapor concentration in the SVT.

Figure 7 shows the dependences of the EQE spectra and the current-voltage characteristics under AM1.5 (100 mW/cm^2^) solar-illumination of the solar cells on the vapor concentration and temperature while exposing the interpenetrating film to solvent vapor. The performance parameters of the solar cells with and without SVT are summarized in Table 1 and Table 2. The EQE increased as a result of SVT, depending on the vapor concentration and temperature. Judging from the photovoltaic properties, the optimized conditions of SVT appear to be 21.6 vol % and 25 °C at this stage. In comparison with the solar cell without SVT, the solar cell with SVT demonstrated an improvement of *V*_oc_ and FF from 0.30 to 0.37 V and from 0.31 to 0.49, respectively. However, excess introduction of the solvent vapor deteriorates the photovoltaic characteristics of the solar cell. From the results described above, it is considered that optimization of the solvent vapor pressure inside the petri dish is one of the key factors leading to the improvement of solar cell performance. Further optimization of the SVT conditions, such as solvents and external fields, is still necessary for achieving higher performance solar cells.

**Table 1:** Performance parameters in current-voltage characteristics of the solar cells under AM1.5 solar-illuminated conditions depending on the vapor concentration at 25 °C in the SVT.

Vapor concentration [vol %]	*I_sc_* [mA/cm_2_]	*V_oc_* [V]	Fill Factor	η_e_ [%]
w/o treatment	4.45	0.30	0.31	0.54
13.0	4.23	0.39	0.41	0.68
21.6	4.19	0.37	0.49	0.76
30.3	4.12	0.31	0.36	0.46

**Table 2:** Performance parameters in current-voltage characteristics of the solar cells under AM1.5 solar-illuminated conditions depending on vapor temperature at 21.6 vol % vapor concentration in the SVT.

Vapor temperature [°C]	*I*_sc_ [mA/cm^2^]	*V*_oc_ [V]	Fill Factor	η_e_ [%]
w/o treatment	4.45	0.30	0.31	0.54
15	3.01	0.41	0.33	0.56
25	4.19	0.37	0.49	0.76
50	4.87	0.32	0.39	0.60

## 4. Conclusion

The effects of SVT on a PAT6 thin film and its application to interpenetrating heterojunction organic solar cells were investigated. Upon introducing the SVT process, the crystallinity of the PAT6 film was improved, and the hole mobility and conductivity of spin-coated PAT6 thin films were enhanced. It was confirmed that SVT was also effective for improving the crystallinity and surface morphology of the C_60_/PAT6 interpenetrating layer from the results of the absorption spectra and SEM surface observation. Solar cells that underwent SVT at an appropriate solvent concentration and temperature exhibited a higher EQE, *V*_oc_, and FF than untreated solar cells.
